# Assessing Predictive Factors for Thrombectomy Necessity in Acute Ischemic Stroke: Insights From the Direct to Angio Suite Protocol

**DOI:** 10.1161/SVIN.124.001476

**Published:** 2026-05-05

**Authors:** Whitfield Lewis, Keiko A. Fukuda, Shashvat M. Desai, Cynthia L. Kenmuir, Mohamed Shehab-Eldin, Mausaminben Y. Hathidara, Ashutosh P. Jadhav

**Affiliations:** 1 Division of Cerebrovascular Medicine, University of Pittsburgh Medical Center (UPMC) Altoona, UPMC Neurological Institute-Stroke Center, PA (W.L., C.L.K., M.S.-E., M.Y.H.).; 2 Department of Neurology, University of Pittsburgh School of Medicine, PA (W.L., C.L.K., M.S.-E., M.Y.H.).; 3 Department of Neurology, University of California, San Francisco (K.A.F.).; 4 Neuroscience Research Division (Neurovascular Program), HonorHealth Research and Innovation Institute, Scottsdale, AZ (S.M.D.).; 5 Department of Neurology and Neurosurgery, Barrow Neurological Institute, Phoenix, AZ (A.P.J.).

**Keywords:** ischemic stroke, morbidity, reperfusion, retrospective studies, thrombectomy

## Abstract

**BACKGROUND:**

Endovascular thrombectomy (EVT) has emerged as an effective treatment option. Unfortunately, interfacility transfers to thrombectomy-capable centers often delay treatment time. The direct to angio suite (DTA) pathway reduces interfacility transfer time by foregoing the emergency department at the thrombectomy-capable center and directing patients immediately to the angio suite. Up to 22% of patients transferred under the DTA pathway will not have a large vessel occlusion on cerebral angiography or will experience significant neurological improvement, obviating the need for EVT. This study evaluated predictive factors associated with EVT under the DTA protocol.

**METHODS:**

A retrospective review was conducted on 2590 patients transferred to 2 comprehensive stroke centers from January 2015 to December 2020. Data from 407 patients transferred under the DTA protocol were analyzed to identify factors associated with undergoing EVT using multivariable logistic regression adjusted for age, sex, National Institutes of Health Stroke Scale (NIHSS) score on arrival at the comprehensive stroke center, Alberta Stroke Program Early Computed Tomography Score, decrease in NIHSS score from initial presentation to arrival at the comprehensive stroke center by ≥5 points (change in NIHSS score from referring hospital to comprehensive stroke center), intravenous thrombolytics, and computed tomography angiography–confirmed large vessel occlusion. Statistical analysis included the Mann-Whitney *U* test, the Fisher exact test, the Pearson χ^2^ test, and the logistic regression analysis.

**RESULTS:**

Of the 407 patients transferred under the DTA protocol (median age, 73 [interquartile range, 63–82] years; 50% female), 355 underwent EVT, while 52 did not. Among patients who did not undergo EVT, 12 demonstrated significant neurological improvement on arrival, obviating the need for intervention. Among the remaining 40 patients, diagnostic cerebral angiography demonstrated distal clot migration or patent vessels, precluding thrombectomy. Factors associated with EVT included comprehensive stroke center NIHSS score, large vessel occlusion on computed tomography angiography at the satellite hospital, change in NIHSS score from referring hospital to comprehensive stroke center score, intravenous thrombolytics, and Alberta Stroke Program Early Computed Tomography Score. Comprehensive stroke center NIHSS score ≥8 (odds ratio, 5.99) and large vessel occlusion on computed tomography angiography (odds ratio, 5.36) were associated with higher odds of EVT. In contrast, change in NIHSS score from referring hospital to comprehensive stroke center score (odds ratio, 0.38), intravenous thrombolytics (odds ratio, 0.41), and Alberta Stroke Program Early Computed Tomography Score ≥9 (odds ratio, 0.22) were associated with lower odds of EVT. Tenfold cross-validation demonstrated a sensitivity of 96.65%, positive predictive value of 89.15%, and negative predictive value of 65.22%. The full model demonstrated strong discrimination (area under the curve, 0.84).

**CONCLUSIONS:**

The study identifies key predictors of EVT under the DTA protocol, highlighting the role of initial clinical assessment and neuroimaging in optimizing patient selection and reducing unnecessary interventions.

## GRAPHIC ABSTRACT

**Figure:**
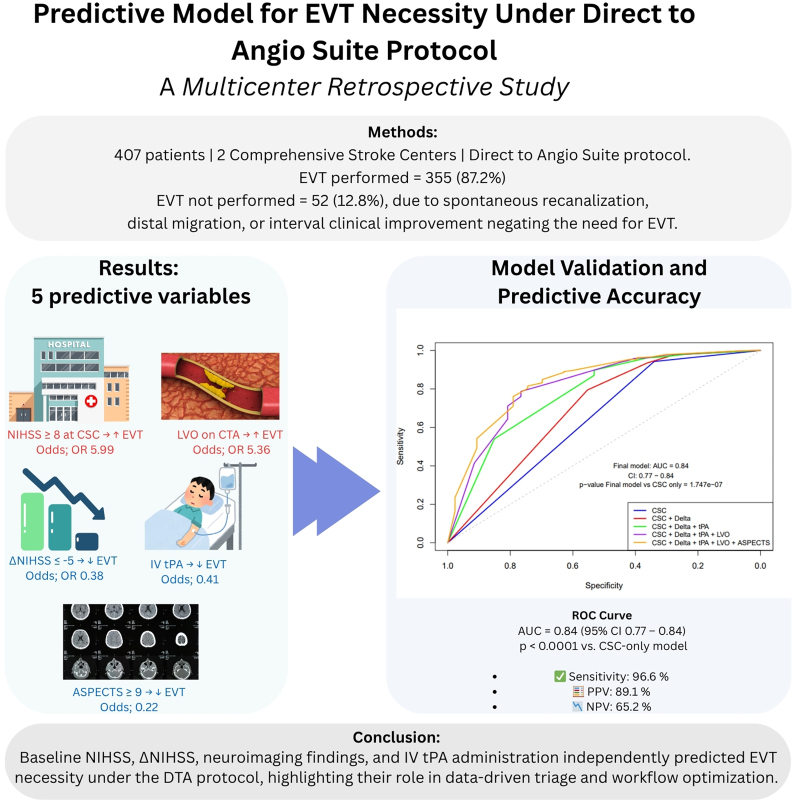

**CLINICAL PERSPECTIVE**

**What Is New?**

- The direct to angio suite protocol reduces door-to-groin-puncture time in acute ischemic stroke, but broad selection criteria can lead to unnecessary transfers and groin punctures.
- This study identified 5 independent predictors (comprehensive stroke center National Institutes of Health Stroke Scale score ≥8, change in National Institutes of Health Stroke Scale score from referring hospital to comprehensive stroke center ≤–5, large vessel occlusion on computed tomography angiography, intravenous thrombolytics administration, and Alberta Stroke Program Early Computed Tomography Score ≥9) that were associated with the likelihood of undergoing endovascular thrombectomy within a direct to angio suite workflow, achieving an area under the curve of 0.84 with high sensitivity on 10-fold cross-validation.

**What Are the Clinical Implications?**

- A future thrombectomy-likelihood score, built on these predictive variables and expanded as larger data sets become available, could guide direct to angio suite transfer decisions and optimize angiography resource use during high-demand scenarios.

Early reperfusion therapies are effective in mitigating morbidity and mortality in the setting of acute ischemic stroke.^1^ The landmark intravenous thrombolytic trials of 1995 and 1997 demonstrated that earlier reperfusion is associated with improved functional outcomes, whereas delays in reperfusion are associated with worse outcomes.^1^ Since then, significant resources and research time have gone into the refinement of workflow protocols with the aim of improving reperfusion time.^1^ Some limitations noted from the intravenous thrombolytic trials included the low rate of recanalization in large vessel occlusions (LVOs) and the lack of evidence-based benefit in patients presenting after 4.5 hours of symptom onset.^1,2^ Due to the reduced efficacy of intravenous thrombolytics for LVOs, endovascular thrombectomy (EVT) for LVOs was explored. In 2015, pooled data from 5 randomized controlled trials showed a clear outcome benefit of EVT for acute ischemic stroke with a low number needed to treat of 2.6 to reduce disability, defined by a reduction in the modified Rankin Scale score by at least 1 point.^3^ Later in 2018, the DAWN trial (DWI or CTP Assessment With Clinical Mismatch in the Triage of Wake‐Up and Late Presenting Strokes Undergoing Neurointervention With Trevo) was able to extend the treatment time up to 24 hours in a select cohort of patients with stroke with patients treated earlier in the time window having better functional outcome than those treated later in the time window.^4^

There is an estimate of 19 500 to 32 000 thrombectomy-eligible patients in the United States yearly. Despite this large number, there are only 262 comprehensive stroke centers (CSCs) and an additional 44 thrombectomy-capable stroke centers.^5^ A recent review of data from hospitals in 11 states revealed that less than half of patients with acute ischemic stroke arrived initially at a thrombectomy-capable stroke center; interfacility transfer is, therefore, paramount in providing patients’ access to necessary mechanical reperfusion care.^5,6^ Unsurprisingly, patients undergoing interfacility transfer to a thrombectomy-capable center tend to have an increase in time from symptom onset to EVT, with the SWIFT PRIME (Solitaire With the Intention for Thrombectomy as Primary Endovascular Treatment) data showing more than a 90-minute increase in time for mechanical reperfusion.^7^

Improvement in the workflow of interfacility transfers has now become a focus in the acute stroke treatment paradigm. Bypassing the emergency department at thrombectomy-capable centers and directing the acute patient immediately to the angio suite for mechanical reperfusion have been shown to significantly reduce the time from symptom onset to reperfusion. While a direct to angio suite (DTA) approach can improve stroke care in the right patient population, overtransfer can overwhelm these centers. A 2019 retrospective cohort study demonstrated that 15% of patients involved in interfacility transfer had a significant improvement in National Institutes of Health Stroke Scale (NIHSS) score, sufficient to obviate the need for EVT.^8^ Another 2019 study evaluating patients transferred to a CSC for EVT showed that 40.6% of these patients had patent vessels or distal occlusions upon arrival to the CSC, despite an average NIHSS score of 9.^9^ Our current study examined predictors of EVT in patients transferred under the DTA protocol.

## Methods

### Study Design and Setting

This study followed the Strengthening the Reporting of Observational Studies in Epidemiology guidelines (see Checklist in the Supplemental Material). The study was approved by the local institutional review board at the University of Pittsburgh Medical Center. Consecutive prospective cases transferred to 2 CSCs were retrospectively reviewed from January 2015 to December 2020 using the institutional Get With The Guidelines-Stroke database.

The telestroke system operates 24 hours a day and 365 days a year, and our institution coordinates transfers from satellite hospitals (outside hospital [OSH]) to our CSCs. Consults are first placed to a vascular neurology fellow under the supervision of a board-certified vascular neurologist. Patients are evaluated using a video monitor, with review of baseline noncontrast head computed tomography and computed tomography angiography (CTA), neurological examination, and clinical history.

If findings indicate acute ischemic stroke due to an LVO, consideration is made for transfer to one of our CSCs.

### Patient Selection and Eligibility

Patients were assessed for eligibility for the DTA pathway. Criteria for DTA pathway inclusion included last seen well ≤6 hours, NIHSS score ≥9, Alberta Stroke Program Early Computed Tomography Score (ASPECTS) ≥6, and the presence of an intracranial LVO. LVOs included intracranial internal carotid artery (ICA) occlusion; first or second branch middle cerebral artery occlusion (M1 or M2); first or second branch anterior cerebral artery occlusion (A1 or A2); and basilar artery occlusion. Patients were directed toward the DTA pathway once these criteria were met.

Intravenous thrombolytic timing was determined by the last seen well time and recorded administration time as documented in the patient's chart. LVO status was determined by the interpreting stroke fellow and attending on the telestroke call, with findings documented in the chart. When this information was missing, CTA images were independently reviewed by the author and coauthor to determine the presence and location of LVO.

Diagnostic cerebral angiography outcomes, including confirmation of patent vessels versus persistent or distal occlusion, were abstracted from the neurointerventional procedure note documented in the electronic medical record. When documentation was incomplete or ambiguous, angiographic images were reviewed by the author and coauthor to adjudicate the final outcome.

### Data Availability Statement

Deidentified data supporting this study’s findings are available from the corresponding author upon reasonable request, in compliance with institutional policies.

### Statistical Analysis

A *P* value of 0.05 was set as the threshold for statistical significance. The Mann-Whitney *U* test was used to assess differences between groups for continuous variables. Results were reported as medians and interquartile ranges or mean and SD. Categorical variables were expressed as percentages or proportions, with the Fisher exact test and the Pearson χ^2^ test used for group comparisons.

Variables that showed association with the outcome variable were selected for inclusion into the logistic regression model if the significance of the *P* value was ≤0.10. Logistic regression analysis was performed, incorporating both forward selection and backward elimination. Variables with a *P* value of <0.05 were retained in the final model. To address multicollinearity, the variance inflation factor was calculated for each variable in the final model, with a threshold of variance inflation factor ≥5, indicating significant multicollinearity. Variables meeting the threshold of a variance inflation factor of 5 were excluded from the final model. Sensitivity analysis further refined the model, considering AIC and log-likelihood when selecting the best-fitting model. Key predictors were then binarized a priori using clinically relevant thresholds supported by prior literature: CSC NIHSS score was dichotomized at ≥8 versus <8 to reflect more severe presentations and higher likelihood of EVT; change in NIHSS score from referring hospital to comprehensive stroke center (Delta NIHSS) score at ≤–5 versus >–5 to capture early neurological improvement; and OSH ASPECTS at ≥9 versus <9 to reflect smaller versus larger ischemic cores. These thresholds were chosen to improve clinical interpretability while maintaining model performance.

The final logistic regression model underwent several goodness-of-fit tests to ascertain its overall statistical significance (Table S1). These included pseudo R-square measures for explained variance, the likelihood ratio test for model comparison, and receiver operating characteristic curve analysis with area under the curve testing to evaluate the model’s discriminative ability. K-fold cross-validation was then used to generate a predictive model, determining its sensitivity, specificity, positive predictive value, and negative predictive value for predicting thrombectomy needs in new patients. Statistical analysis was performed using R software, version 4.2.3.

## Results

**Figure 1.**
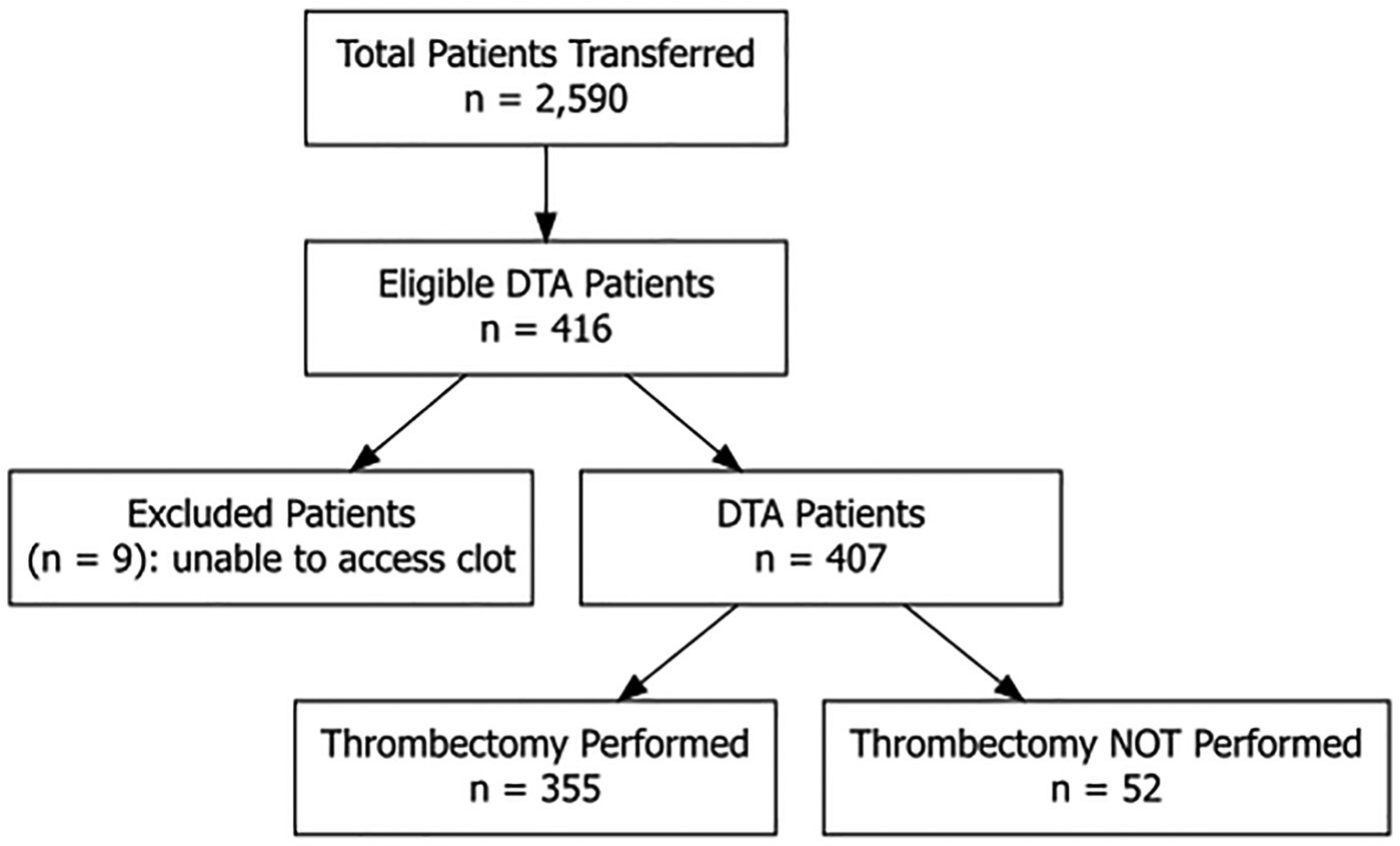
**Flowchart of patients transferred from outside hospitals under the direct to angio suite (DTA) protocol between 2015 and 2020.** Eligible patients were assessed for large vessel occlusion, with exclusions for technical inability to access the clot.

A total of 2590 patients were transferred from OSHs to one of our CSCs between January 2015 and December 2020 (Figure 1). Of these, 1673 patients underwent EVT for acute LVO. In total, 355 patients receiving EVT were transferred under the DTA pathway. A total of 917 transferred patients did not undergo EVT, and chart review of these patients was performed to determine how many were transferred under the DTA pathway. Sixty-one patients were transferred under the DTA pathway but did not undergo EVT on arrival. Nine of these patients still had a high NIHSS score and an LVO, but technical difficulties precluded EVT (eg, difficult access due to tortuosity of the cerebrovascular system). These 9 patients were excluded from the final cohort. The final cohort consisted of 407 patients transferred under the DTA protocol, including 355 who underwent EVT and 52 who did not due to symptom improvement or resolution and patent vessels on diagnostic cerebral angiography.

We evaluated the association between 23 candidate variables and receipt of EVT among patients transferred under the DTA protocol (Table 1). Variables assessed included age, sex, body mass index, race, time from last known well to intravenous thrombolytics, time from last known well to angio suite arrival, night shift (6:00 pm–6:00 am), weekend shift (Friday 6:00 pm–Monday 6:00 am), wake-up stroke, stroke subtype, seizure history, known atrial fibrillation, recent anticoagulation use, ASPECTS at the OSH, NIHSS score at the OSH, NIHSS score at the CSC, Delta NIHSS score, mode of transport to the CSC (air versus ground), CTA performed at the OSH, presence of LVO on OSH CTA, tandem occlusion, intracranial ICA occlusion, M1 occlusion, M2/M3 occlusion, and basilar artery occlusion.

**Table 1.**
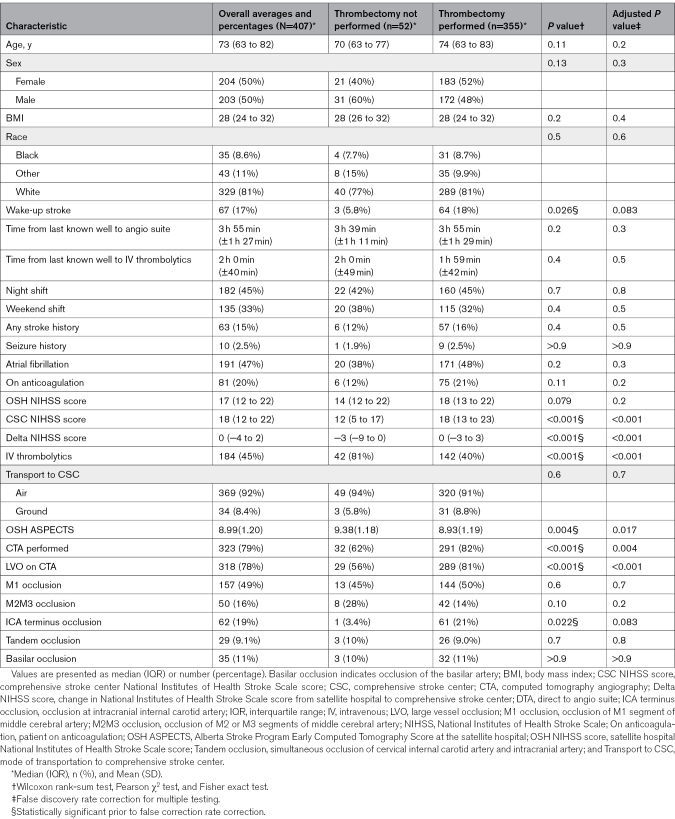
Baseline Characteristics of Patients Transferred Under the DTA Protocol, Stratified by Thrombectomy Status

Initial univariable analyses demonstrated statistically significant associations between several clinical and imaging variables and receipt of EVT. Patients who underwent thrombectomy were more likely to present as wake-up strokes (18% versus 5.8%), less likely to have received intravenous thrombolytics (40% versus 81%), and had higher NIHSS scores at both the OSH (median, 18 versus 14) and CSC (median, 18 versus 12). These patients also had a higher prevalence of ICA terminus occlusion (21% versus 3.4%), greater neurological improvement during transfer as reflected by Delta NIHSS score (median, 0 versus −3.3), more frequent performance of CTA at the OSH (79% versus 62%), and more frequent detection of LVO on OSH CTA (78% versus 56%). ASPECTS (8.93 versus 9.38) and the proportion of M2 occlusions (16% versus 28%) were lower in the thrombectomy group though the difference in M2 occlusions did not reach statistical significance.

Multivariable logistic regression identified 5 independent predictors associated with the performance of EVT under the DTA protocol (Table 2). CSC NIHSS score ≥8 was associated with increased odds of thrombectomy (odds ratio [OR], 5.99). The presence of an LVO on CTA at the OSH was also associated with higher odds of thrombectomy (OR, 5.36). In contrast, early neurological improvement during transfer, defined as a Delta NIHSS score ≤−5, was associated with reduced odds of thrombectomy (OR, 0.38). Administration of intravenous thrombolytics was similarly associated with lower odds of thrombectomy (OR, 0.41). Higher ASPECTS (≥9) was associated with reduced odds of thrombectomy compared with ASPECTS <9 (OR, 0.22).

**Table 2.**
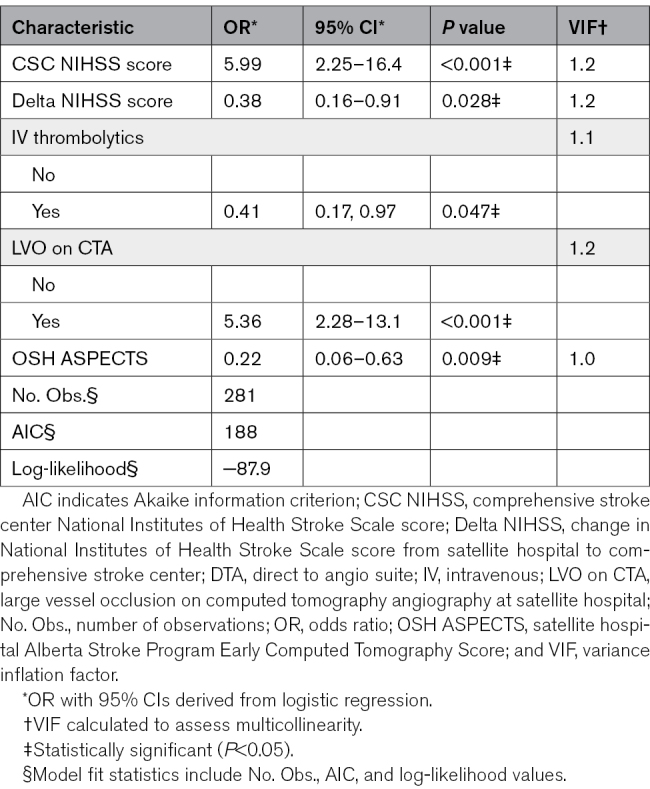
Odds Ratios of Variables Predicting Thrombectomy Performance Under the DTA Protocol, Using Logistic Regression Analysis

The final model demonstrated good discriminative performance, with an area under the receiver operating characteristic curve of 0.84. Tenfold cross-validation yielded a sensitivity of 96.65%, a positive predictive value of 89.15%, and a negative predictive value of 65.22%.

## Discussion

In this retrospective series, 12.8% of patients (52 patients) transferred under the DTA protocol did not undergo thrombectomy (thrombectomy was not attempted). In this 52-patient cohort, 17% of patients bypassed the angio suite entirely due to significant neurological improvement upon arrival at the CSC, avoiding groin puncture and diagnostic angiography. Of the remaining patients in the 52-patient cohort, 83% underwent groin puncture and diagnostic angiography; however, thrombectomy-specific resources were not ultimately utilized in these cases. The most commonly cited reasons for aborting thrombectomy in this group were patent vessel or distal vessel occlusion in 86% of cases, subocclusive thrombus in 7% of cases, and clinical improvement in the remaining 7% of cases (Table S2).

A key finding is that 81% of the 52-patient cohort received intravenous thrombolytics compared with just 41% in the thrombectomy group, emphasizing a 2-fold higher administration rate of thrombolytics in the 52-patient cohort. Furthermore, we observed a striking difference in the occlusion sites: there were twice as many M2 occlusions on the initial CTA of the 52-patient cohort compared with the thrombectomy group. Most notably, the prevalence of ICA terminus occlusion in the 52-patient cohort was only 3.4% compared with 21% in the thrombectomy cohort, representing a 6-fold increase in ICA terminus occlusion in the thrombectomy group. This difference is highly relevant, as supported by a retrospective study on patients with LVO treated with intravenous thrombolytics, which demonstrated that the recanalization rate after intravenous thrombolytics was significantly lower in patients with ICA terminus occlusion (4.4%) compared with those with M2 occlusions (30.8%).^2^ This evidence aligns with our findings and underscores the importance of occlusion location in determining the likelihood of early recanalization. Thus, the higher rate of intravenous thrombolytic administration in the 52-patient cohort, the much higher prevalence of M2 occlusion, and the considerably lower prevalence of ICA terminus occlusion contributed to a markedly higher rate of early recanalization in this cohort. Consequently, early neurological improvement was profound; 17% of the patients in this cohort were deferred from a trip to the angio suite due to their low NIHSS scores. Furthermore, the overall NIHSS score at 24 hours was significantly lower in this cohort compared with the thrombectomy group, with 81% of patients in the 52-patient cohort being discharged to home or inpatient rehabilitation, compared with only 66% of thrombectomy patients (Table 3).

Overall, intravenous thrombolytics were administered in 184 patients of the 407-patient cohort but deferred EVT in only 42 patients (23%). Thirty-seven of these 42 patients had diagnostic cerebral angiogram or follow-up CTA/magnetic resonance angiography showing complete recanalization in 11 patients and distal occlusion in 19 patients (Table S3). Although the rate of recanalization observed in our study is higher than reported in a recently published 2023 study, it remains relatively unimpressive.^2^ This modest rate of recanalization after intravenous thrombolytics continues to challenge the utility of bridging therapy, which is the concomitant use of intravenous thrombolytics in patients on their way to the angio suite for EVT. In a recent 2021 study involving patients at our stroke institution, bridging therapy with patients arriving in the emergency department resulted in a greater delay in door-to-puncture time without improving the overall rate of recanalization compared with patients bypassing intravenous thrombolytics for direct EVT.^10^ However, our 407-patient cohort was invariably transferred from an outside facility, and neither the time from last seen well to angio suite nor the rate of hemorrhagic complication was adversely affected by intravenous thrombolytics. In addition, while a meta-analysis of randomized controlled trials on bridging therapy versus direct EVT showed less than a 30-minute gap between intravenous thrombolytic administration and thrombectomy, our cohort had a 2-hour gap.^11^ This extended time may have allowed for intravenous thrombolytics to take effect more fully, leading to the observed relatively high rate of early recanalization in our cohort. Importantly, the proportion of M2 occlusions in this meta-analysis was lower (9%) compared with our cohort (13%).^11^ Conversely, the proportion of ICA terminus occlusions in this meta-analysis was higher at 27%, while our cohort had a lower rate of ICA terminus occlusion (20%).^11^ This meta-analysis concluded that bridging therapy did not improve functional outcome, but this discrepancy in occlusion sites likely played a crucial role in the different outcome.^11^ Intravenous thrombolytic administration was a strong independent factor in reducing the need for groin punctures and diagnostic cerebral angiograms. Bridging therapy in the context of the DTA protocol remains a vital tool in the armamentarium of acute ischemic stroke care.

Surprisingly, the OSH NIHSS score failed to show an association with the performance of EVT. The median NIHSS score at OSH was 18 for EVT patients and 14 for non-EVT, but this was not statistically significant. The smaller sample size (52 patients) in the non-EVT group could have resulted in underpowering of the study regarding this statistical outcome. As new data become available, this point can be further explored. When intravenous thrombolytics are considered for treatment, the patient is evaluated by a vascular-trained neurologist at the telemedicine service. The NIHSS score assessed at this time is likely accurate, and previous studies have demonstrated high interrater reliability of the NIHSS score in this context.^12^ Conversely, in cases where intravenous thrombolytics are not considered, the initial NIHSS score may not have been performed by well-trained personnel, resulting in inaccuracies in the OSH NIHSS score. Likewise, the association between OSH NIHSS score and early recanalization has been inconsistent in the stroke literature. Higher NIHSS score at the OSH has been shown to be positively associated with non-EVT DTA transfers, while another large series showed no association.^13,14^

On the other hand, a lower CSC NIHSS score was strongly associated with a decreased likelihood of EVT performance in our DTA cohort. Patients who had an NIHSS score <6 upon arrival to the CSC did not even undergo groin puncture or diagnostic angiogram despite the presence of an LVO at the OSH CTA in 89% of cases (3 patients had only subocclusive thrombus) and despite the NIHSS score at the OSH being as high as 17. In the 52 patients in our cohort that did not receive EVT, all 9 of the patients who did not undergo groin puncture presented with an NIHSS score <6 at our CSC. All 9 patients received intravenous thrombolytics, once again underscoring the importance of bridging therapy in reducing the amount of unnecessary groin punctures and diagnostic cerebral angiograms. Of the remaining 43 non-EVT patients who did undergo groin puncture and diagnostic cerebral angiogram, the average NIHSS score on arrival at the CSC was 14. In these 43 patients, the Delta NIHSS score was −3, only 53% had CTA performed at the OSH, intravenous thrombolytics were administered at a high rate (77%), and most of these patients had an ASPECTS of 10 (64%). On diagnostic cerebral angiogram for these 43 patients, the most common angiographic finding was distal occlusion (49%) or completely patent vessels (37%). Despite a much higher CSC NIHSS score in this cohort of 43 patients, the collective influence of the other predictive variables (intravenous thrombolytics, Delta NIHSS score, CTA at OSH, and ASPECTS) on deferral of EVT was profound. After conducting sensitivity analysis and adjusting for other predictive variables, a CSC NIHSS score of ≥8 highly predicted the need for EVT when a cerebral angiogram was done, while a score of <8 strongly indicated a lower likelihood of requiring EVT.

There was a statistically significant difference between the Delta NIHSS score in patients without EVT versus patients with EVT (−3 versus 0), and the Delta NIHSS score was an independent predictor of EVT in our model. A 2022 study by Terreros et al^14^ found that an average Delta NIHSS score of −5 was associated with early recanalization of patients transferred for EVT. A recent 2023 systematic review and meta-analysis by Kobeissi et al^15^ demonstrated that a range between −4 and −8 is usually deemed to be early neurological improvement. Appropriately, the best-fitting and the most predictive Delta NIHSS score for our model was found to be −5. Patients with a Delta NIHSS score of −5 or lower (ie, −6 and −7, indicating early neurological improvement) were significantly more likely to have early recanalization or distal occlusion when a diagnostic angiogram was performed.

In our study, we used ASPECTS, the location of LVO, the performance of CTA at OSH (CTA performance), and LVO detection on CTA at the OSH to analyze neuroimaging associations with EVT. ASPECTS, CTA performance, and LVO on CTA were strong predictors for EVT. Although intracranial ICA occlusion (mainly ICA terminus) had a statistically significant association with bivariate analysis, the association was no longer statistically significant when included in the logistic regression model, and the overall fit of the model was reduced by its inclusion. CTA performance at the OSH was similar to LVO detection at the OSH, but there were 3 patients who had subocclusive thrombus without LVO on CTA at the OSH, 1 patient with cervical stenosis and distal occlusion at the OSH, and 1 patient whose image could not be interpreted due to motion artifact. As well, CTA performance and LVO detection on CTA showed significant multicollinearity with logistic regression analysis. The removal of CTA performance from the final model allowed for a better-fitting model. Finally, an ASPECTS of ≥9 was also a strong independent predictor for non-EVT. This is consistent with previous studies and likely reflects a smaller ischemic core and better collateral flow to the ischemic territory.^16^

Notably, the proportion of basilar artery occlusions was similar between patients who ultimately underwent thrombectomy and those who did not (11% versus 10%). Although this proportion is toward the higher end of reported ranges, it remains consistent with contemporary series of posterior circulation LVO. Posterior circulation strokes present unique clinical considerations, as traditional decision tools, such as NIHSS score and ASPECTS, have recognized limitations in predicting tissue viability and clinical trajectory in basilar artery occlusion. Given the catastrophic natural history associated with untreated basilar artery occlusion, including high risks of mortality and severe disability, clinicians may appropriately maintain a lower threshold for interfacility transfer, angiographic evaluation, and consideration of endovascular therapy, even when early neurological improvement or imaging features might otherwise argue against intervention.^17^

After finalizing the logistic regression model, we used multiple validation techniques to assess its validity and predictive accuracy. The model’s goodness-of-fit was evaluated using pseudo R-square measures. Specifically, a Nagelkerke R-square value of 0.3572 indicated a moderate level of variance explained by the model, while a McFadden R-square of 0.2686 suggested an acceptable fit with the observed data. The likelihood ratio test, comparing the full logistic regression model to a reduced model, was statistically significant, yielding a χ^2^ statistic of 64.61 and a *P* value of <0.0001. These results collectively underscore the robustness and reliability of our model within the study’s context.

**Figure 2.**
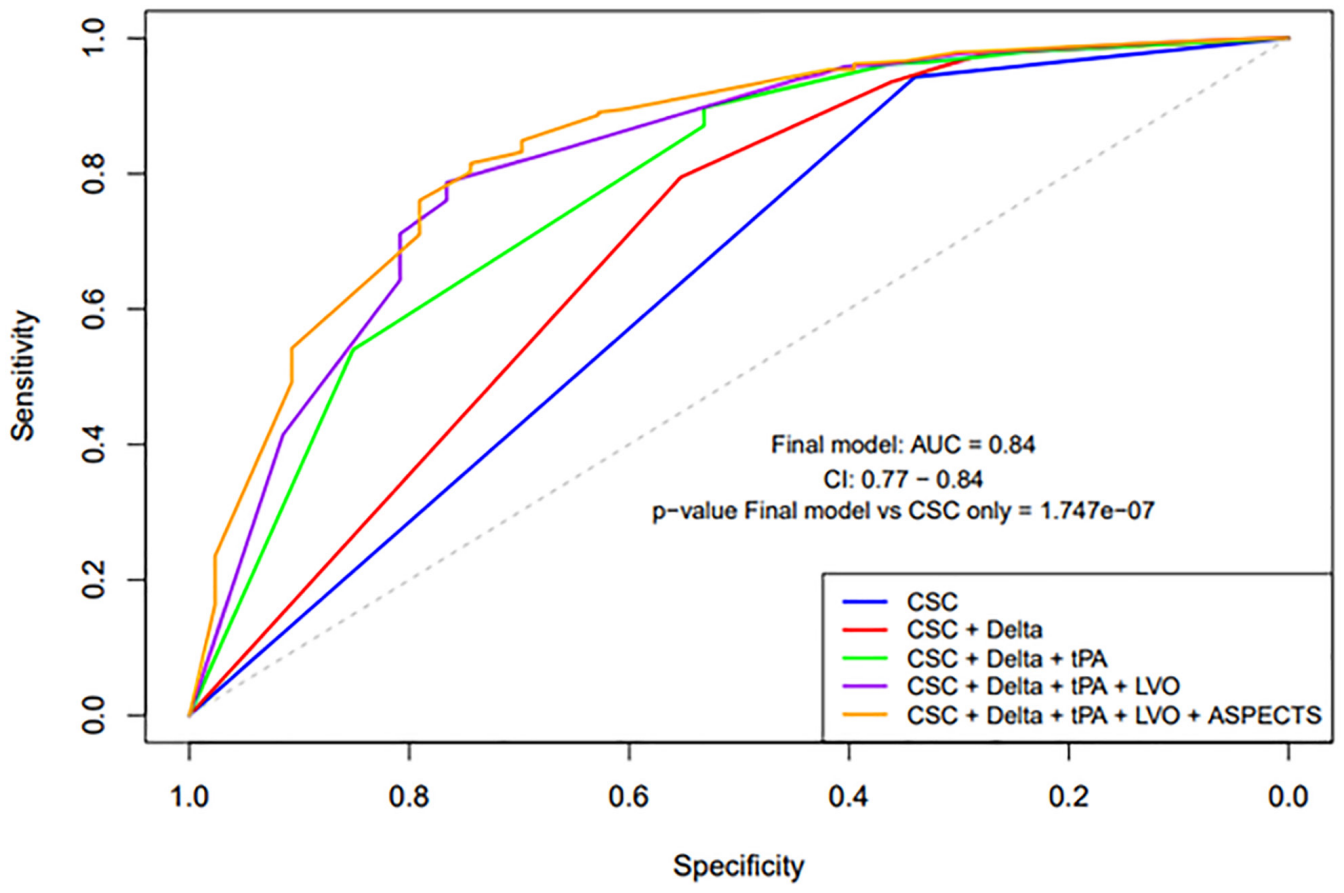
**Receiver operating characteristic curves for 5 logistic regression models predicting thrombectomy performance under the direct to angio suite protocol.** Models included comprehensive stroke center (CSC) National Institutes of Health Stroke Scale (NIHSS) score alone; CSC NIHSS score with change in NIHSS score from outside hospital to CSC (Delta NIHSS score); CSC NIHSS score with Delta NIHSS score and intravenous (IV) thrombolytics; CSC NIHSS score with Delta NIHSS score, IV thrombolytics, and large vessel occlusion (LVO) at outside hospital CTA; and the full model including CSC NIHSS score, Delta NIHSS score, IV thrombolytics, LVO at outside hospital CTA, and Alberta Stroke Program Early Computed Tomography Score (ASPECTS). Area under the curve (AUC)=0.84 (95% CI, 0.77–0.84).

Subsequently, k-fold cross-validation was conducted to evaluate the model’s ability to generalize and accurately predict thrombectomy needs in new, unseen data. This validation process resulted in a high sensitivity of 96.64% for detecting patients who will require thrombectomy in the DTA protocol, demonstrating the model’s strong capability in correctly identifying true positive cases. In addition, the model showed a positive predictive value of 89.15%, suggesting a high likelihood of accurate thrombectomy predictions, and a negative predictive value of 65.22%, indicating reasonable accuracy in predicting when thrombectomy is not needed. This comprehensive view of the model’s applicability and reliability in real-world scenarios is further illustrated in Figure 2, which displays a receiver operating characteristic curve plotting true positives against false positives. This curve reveals a strong area under the curve of 0.84, signifying a significant enhancement in the model’s accuracy with the inclusion of all 5 predictive variables. Figure 2 also shows a *P* value of significance <0.0001 when comparing the final model with all 5 variables included compared with a more basic model that included only 1 variable.

Outcomes between the 2 groups differed based on discharge facility, discharge NIHSS score, and follow-up modified Rankin Scale score (Table 3). Rates of symptomatic intracerebral hemorrhage and malignant middle cerebral artery syndromes were similar between groups.

**Table 3.**
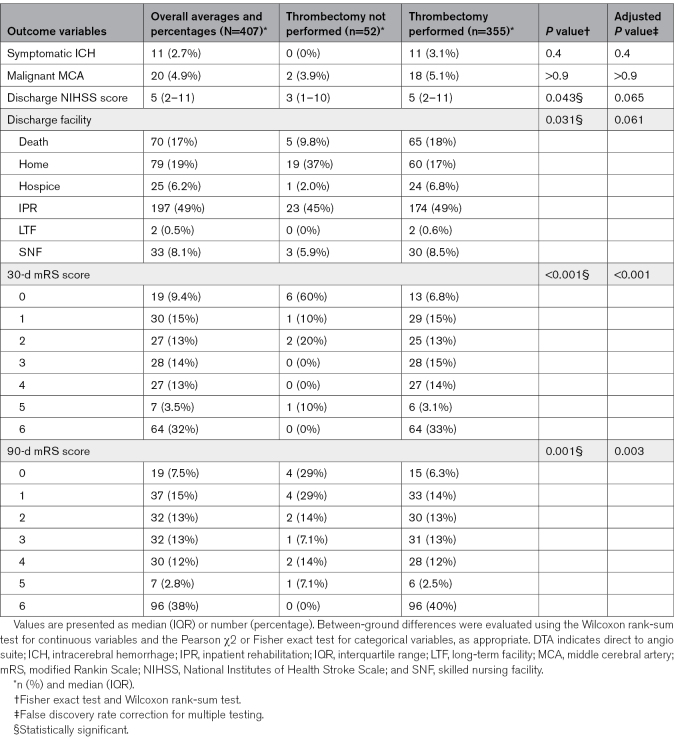
Discharge and Follow-Up Outcomes of Patients Transferred Under the DTA Protocol, Stratified by Thrombectomy Status

This study has several limitations. First, its retrospective design introduces potential selection and documentation biases inherent to chart review and registry-based analyses. Second, because transfers were only initiated when an LVO was identified at the OSH, patients with false-negative CTA interpretations were excluded from our analytic cohort. Although CTA studies were interpreted by telestroke providers in conjunction with OSH radiologists, subtle or distal occlusions may have been missed. If such patients had been correctly identified and transferred, some would likely have undergone EVT and entered the EVT cohort, while others might have experienced spontaneous recanalization or distal migration and been classified in the no-EVT cohort. Without prospective data capturing these missed cases, it is not possible to fully assess their impact on the study’s findings. Third, variability in imaging protocols and interpretation across OSHs may have influenced LVO detection and ASPECTS scoring, despite confirmatory review by the study team when necessary. Finally, as a single-institution analysis, the findings may not be fully generalizable to other health care systems with different telestroke workflows or transfer practices.

In summary, our study offers valuable insights into the predictive factors that can influence thrombectomy decisions under the DTA protocol. The findings highlight the critical role of initial clinical assessment and neuroimaging that can guide treatment strategies, which can assist in optimizing patient outcomes and reducing unnecessary interventions.

## ARTICLE INFORMATION

### Sources of Funding

None.

### Disclosures

Ashutosh P. Jadhav is the Editor-in-Chief for Stroke: Vascular and Interventional Neurology (S:VIN) and was not involved in the handling or final disposition of this article. Disclosures provided by Dr Jadhav in compliance with American Heart Association's annual Journal Editor Disclosure Questionnaire are available at https://www.ahajournals.org/editor-coi-disclosures. Cynthia L. Kenmuir is a Guest Associate Editors for S:VIN and was not involved in the handling or final disposition of this article. Disclosures provided by Dr Kenmuir in compliance with American Heart Association's annual Journal Editor Disclosure Questionnaire are available at https://www.ahajournals.org/editor-coi-disclosures.

### Supplemental Material

Tables S1–S3

## Supplementary Material
