# Supplementary material for: Assessing Predictive Factors for Thrombectomy Necessity in Acute Ischemic Stroke: Insights From the Direct to Angio Suite Protocol
Source: Stroke Vasc Interv Neurol. 2026 May 5;6(3):e001476. doi: 10.1161/SVIN.124.001476 (PMC13189590; doi:10.1161/SVIN.124.001476)
Supplement: Supplementary file 1 [file svi2-6-e001476-s001.pdf]

STROBE Statement—checklist of items that should be included in reports of observational studies

	Item No.	Recommendation	Page No.	Relevant text from manuscript
**Title and abstract**	1	(*a*) Indicate the study's design with a commonly used term in the title or the abstract	Title and Abstract	Retrospective cohort study
		(*b*) Provide in the abstract an informative and balanced summary of what was done and what was found	Abstract	Full abstract summarizes background, methods, results, and conclusion
**Introduction**				
Background/rationale	2	Explain the scientific background and rationale for the investigation being reported	Introduction (last paragraph)	Up to 22% of patients transferred under the Direct to Angio Suite (DTA) pathway will not have a large vessel occlusion on cerebral angiography or will experience significant neurological improvement, obviating the need for EVT… The rationale for this study was to investigate differences in patient characteristics between those who still require thrombectomy after transfer versus those with spontaneous improvement that precludes the need for treatment.
Objectives	3	State specific objectives, including any prespecified hypotheses	End of Introduction	Our current study examined predictors of EVT in patients

				transferred under the DTA protocol.
**Methods**				
Study design	4	Present key elements of study design early in the paper	Methods – Study Design	This retrospective cohort study followed the Strengthening the Reporting of Observational Studies in Epidemiology (STROBE) guidelines… retrospective review of consecutive prospective cases…
Setting	5	Describe the setting, locations, and relevant dates, including periods of recruitment, exposure, follow-up, and data collection	Methods – Patient Selection	two comprehensive stroke centers… January 2015 to December 2020… telestroke system… transfers from satellite hospitals (OSH)
Participants	6	(*a*) *Cohort study*—Give the eligibility criteria, and the sources and methods of selection of participants. Describe methods of follow-up<br>*Case-control study*—Give the eligibility criteria, and the sources and methods of case ascertainment and control selection. Give the rationale for the choice of cases and controls<br>*Cross-sectional study*—Give the eligibility criteria, and the sources and methods of selection of participants	Methods – Patient Selection and Eligibility	last seen well ≤ 6 hours, NIHSS ≥ 9, ASPECTS ≥ 6, and the presence of an intracranial large vessel occlusion… Consecutive prospective cases transferred…
		(*b*) *Cohort study*—For matched studies, give matching criteria and number of exposed and unexposed<br>*Case-control study*—For matched studies, give matching criteria and the number of controls per case		
Variables	7	Clearly define all outcomes, exposures, predictors, potential confounders, and effect modifiers. Give diagnostic criteria, if applicable	Methods + Table 1	CSC NIHSS, delta NIHSS, IV thrombolytics, LVO on CTA,

				OSH ASPECTS (with clinical cutoffs: NIHSS ≥8, delta ≤–5, ASPECTS ≥9)
Data sources/ measurement	8*	For each variable of interest, give sources of data and details of methods of assessment (measurement). Describe comparability of assessment methods if there is more than one group	Methods – Patient Selection	institutional Get With The Guidelines-Stroke database, chart review, telestroke records, neurointerventional procedure notes; images reviewed by authors when ambiguous
Bias	9	Describe any efforts to address potential sources of bias	Discussion – Limitations	retrospective design introduces potential selection and documentation biases… variability in imaging protocols across outside hospitals
Study size	10	Explain how the study size was arrived at	Results (first paragraph)	final cohort consisted of 407 patients transferred under the DTA protocol (all consecutive eligible patients during the study period)

Continued on next page

Quantitative variables	11	Explain how quantitative variables were handled in the analyses. If applicable, describe which groupings were chosen and why	Methods – Statistical Analysis	binarized a priori using clinically relevant thresholds (CSC NIHSS ≥8 vs <8, delta NIHSS ≤–5 vs >–5, OSH ASPECTS ≥9 vs <9)
Statistical methods	12	(*a*) Describe all statistical methods, including those used to control for confounding	Methods – Statistical Analysis	multivariable logistic regression with forward/backward selection, VIF for multicollinearity, 10-fold cross-validation, goodness-of-fit tests
		(*b*) Describe any methods used to examine subgroups and interactions		
		(*c*) Explain how missing data were addressed		
		(*d*) *Cohort study*—If applicable, explain how loss to follow-up was addressed<br>*Case-control study*—If applicable, explain how matching of cases and controls was addressed<br>*Cross-sectional study*—If applicable, describe analytical methods taking account of sampling strategy		
		(*e*) Describe any sensitivity analyses		
**Results**				
Participants	13*	(a) Report numbers of individuals at each stage of study—eg numbers potentially eligible, examined for eligibility, confirmed eligible, included in the study, completing follow-up, and analysed	Results (first paragraph) + Figure 1	2,590 patients transferred → 407 under DTA protocol (355 EVT, 52 no-EVT); 9 technical exclusions
		(b) Give reasons for non-participation at each stage		
		(c) Consider use of a flow diagram		
Descriptive data	14*	(a) Give characteristics of study participants (eg demographic, clinical, social) and information on exposures and potential confounders	Table 1	Baseline characteristics stratified by thrombectomy status (Table 1)
		(b) Indicate number of participants with missing data for each variable of interest		
		(c) *Cohort study*—Summarise follow-up time (eg, average and total amount)		

Outcome data	15*	*Cohort study*—Report numbers of outcome events or summary measures over time	Table 3 + Results	Discharge facility, 30- and 90-day mRS, symptomatic ICH, etc. (Table 3)
		Case-control study—Report numbers in each exposure category, or summary measures of exposure		
		Cross-sectional study—Report numbers of outcome events or summary measures		
Main results	16	(*a*) Give unadjusted estimates and, if applicable, confounder-adjusted estimates and their precision (eg, 95% confidence interval). Make clear which confounders were adjusted for and why they were included	Results + Table 2	Five independent predictors with ORs and 95% CIs (Table 2): CSC NIHSS ≥8 (OR 5.99), LVO on CTA (OR 5.36), etc.
		(*b*) Report category boundaries when continuous variables were categorized		
		(*c*) If relevant, consider translating estimates of relative risk into absolute risk for a meaningful time period		

Continued on next page

Other analyses	17	Report other analyses done—eg analyses of subgroups and interactions, and sensitivity analyses	Results	Goodness-of-fit metrics (Table S1), 10-fold cross-validation (sensitivity 96.65%, AUC 0.84), ROC curves (Figure 2)
**Discussion**				
Key results	18	Summarise key results with reference to study objectives	Abstract + Conclusion	Key predictors identified with strong model performance (AUC 0.84)
Limitations	19	Discuss limitations of the study, taking into account sources of potential bias or imprecision. Discuss both direction and magnitude of any potential bias	Discussion (near end)	Retrospective design, selection bias, single-institution, imaging variability across OSH
Interpretation	20	Give a cautious overall interpretation of results considering objectives, limitations, multiplicity of analyses, results from similar studies, and other relevant evidence	Discussion	Clinical implications for improving DTA patient selection and reducing unnecessary interventions
Generalisability	21	Discuss the generalisability (external validity) of the study results	Discussion (last paragraph)	findings may not be fully generalizable to other healthcare systems with different telestroke workflows
**Other information**				
Funding	22	Give the source of funding and the role of the funders for the present study and, if applicable, for the original study on which the present article is based	Title page	Sources of Funding: None.

*Give information separately for cases and controls in case-control studies and, if applicable, for exposed and unexposed groups in cohort and cross-sectional studies.

Note: An Explanation and Elaboration article discusses each checklist item and gives methodological background and published examples of transparent reporting. The STROBE checklist is best used in conjunction with this article (freely available on the Web sites of PLoS Medicine at http://www.plosmedicine.org/, Annals of Internal Medicine at http://www.annals.org/, and Epidemiology at http://www.epidem.com/). Information on the STROBE Initiative is available at www.strobe-statement.org.
