# Supplementary material for: Assessing Predictive Factors for Thrombectomy Necessity in Acute Ischemic Stroke: Insights From the Direct to Angio Suite Protocol
Source: Stroke Vasc Interv Neurol. 2026 May 5;6(3):e001476. doi: 10.1161/SVIN.124.001476 (PMC13189590; doi:10.1161/SVIN.124.001476)
Supplement: Supplementary file 2 [file svi2-6-e001476-s002.docx]

COVER PAGE

SUPPLEMENTAL MATERIAL

Assessing Predictive Factors for Thrombectomy Necessity in Acute Ischemic Stroke: Insights from the Direct to Angio Suite Protocol

Whitfield Lewis, MD¹²

Keiko A. Fukuda, MD³

Shashvat M. Desai, MD⁴

Cynthia L. Kenmuir, MD, PhD¹²

Mohamed Shehab-Eldin, MD¹²

Mausaminben Y. Hathidara, MD¹²

Ashutosh P. Jadhav, MD, PhD⁵

¹ UPMC Altoona, UPMC Neurological Institute – Stroke Center, Altoona, Pennsylvania, USA

² Department of Neurology, University of Pittsburgh School of Medicine, Pittsburgh, Pennsylvania, USA

³ Department of Neurology, University of California, San Francisco, San Francisco, California, USA

⁴ HonorHealth Research and Innovation Institute, Scottsdale, Arizona, USA

⁵ Department of Neurology and Neurosurgery, Barrow Neurological Institute, Phoenix, Arizona, USA


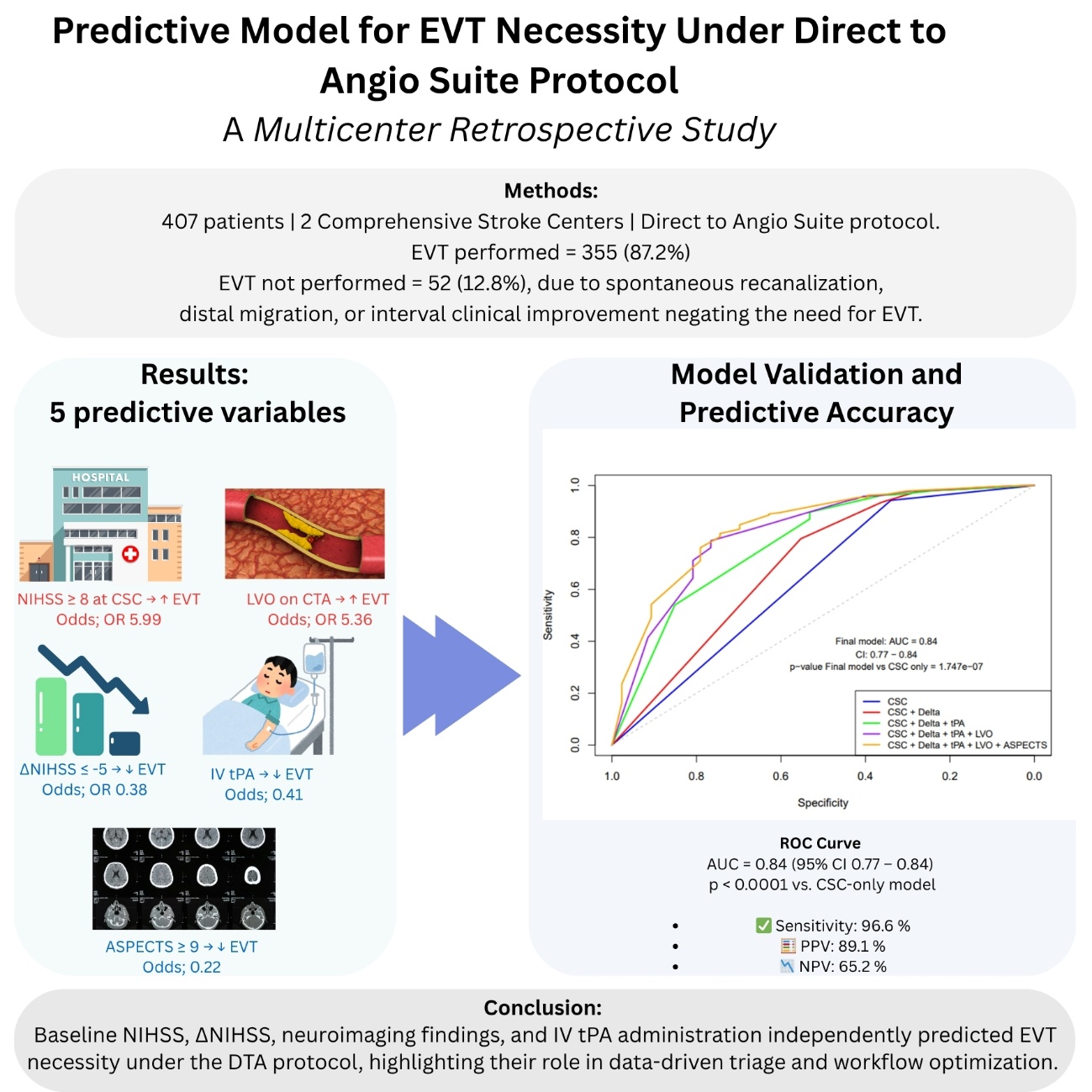


Figure S1. Graphical Abstract summarizing key predictive variables influencing EVT necessity under the Direct to Angio Suite (DTA) protocol.

| **Goodness of Fit Tests** | **Result** | **Model Fit** | **p value** |
| --- | --- | --- | --- |
| McFadden's R-squared | 0.2686 | Good |  |
| Nagelkerke R-squared | 0.3572 | Moderate |  |
| Likelihood Ratio Test | 64.61 | Very Good | < 0.0001 |
| AUC | 0.84 |  |  |
| Sensitivity | 96.64% |  |  |
| Specificity | 34.88% |  |  |
| Positive Predictive Value | 89.15% |  |  |
| Negative Predictive Value | 65.22% |  |  |

Table S1. Goodness-of-Fit Metrics and Performance Summary of the Predictive Model for Thrombectomy Necessity Under the Direct-to-Angio-Suite (DTA) Protocol.

| **Characteristic** | No Groin Puncture, N = 9^1^ | Groin Puncture, N = 43 ^1^ |
| --- | --- | --- |
| **OSH NIHSS** | 12 (11, 13) | 16 (13, 23) |
| **CSC NIHSS** | 4 (2, 4) | 14 (10, 17) |
| **Delta NIHSS** | -8 (-9, -6) | -3 (-7, 1) |
| **OSH ASPECTS** | 9.44(0.73) | 9.36(1.27) |
| **24h NIHSS** | 2 (0, 4) | 9 (4, 17) |
| **IV tPA** | 9 (100%) | 33 (77%) |
| **Time to angio suite** | 3h35min (2h10min,4h10min) | 3h39min (2h42min, 5h19min) |
| **LVO on CTA** | 8 (89%) | 21 (49%) |
| **M1 occlusion** | 6 (67%) | 7 (35%) |
| **M2M3 occlusion** | 1 (11%) | 7 (35%) |
| **ICA terminus occlusion** | 1 (11%) | 0 (0%) |
| **Basilar occlusion** | 0 (0%) | 3 (15%) |
| **LVO confirmed by DSA** |  |  |
| *No* | 4 (67%) | 40 (93%) |
| *Yes* | 1 (17%) | 3 (7.0%) |
| *Yes (done two days later)* | 1 (17%) | 0 (0%) |
| **Thrombectomy performed** |  |  |
| *No* | 9 (100%) | 43 (100%) |
| **Reason for no EVT** |  |  |
| *distal occlusion* | 0 (0%) | 21 (49%) |
| *exam improved* | 9 (100%) | 3 (7.0%) |
| *patent vessels* | 0 (0%) | 16 (37%) |
| *subocclusive* | 0 (0%) | 3 (7.0%) |
| **Discharge Facility** |  |  |
| *Death* | 0 (0%) | 5 (12%) |
| *Home* | 8 (89%) | 11 (26%) |
| *Hospice* | 0 (0%) | 1 (2.4%) |
| *IPR* | 1 (11%) | 22 (52%) |
| *SNF* | 0 (0%) | 3 (7.1%) |
| **30-day mRS** |  |  |
| *0* | 2 (67%) | 4 (57%) |
| *1* | 1 (33%) | 0 (0%) |
| *2* | 0 (0%) | 2 (29%) |
| *5* | 0 (0%) | 1 (14%) |
| **90-day mRS** |  |  |
| *0* | 0 (0%) | 4 (33%) |
| *1* | 2 (100%) | 2 (17%) |
| *2* | 0 (0%) | 2 (17%) |
| *3* | 0 (0%) | 1 (8.3%) |
| *4* | 0 (0%) | 2 (17%) |
| *5* | 0 (0%) | 1 (8.3%) |
| ^1^Median (Q1, Q3); Mean(SD); n (%) | | |

Table S2. Functional Outcomes and Disposition of Patients Not Undergoing Thrombectomy, Stratified by Groin Puncture Status

| **Characteristic** | tPA not given, N = 223^1^ | tPA given, N = 184^1^ | **p-value**^2^ |
| --- | --- | --- | --- |
| **OSH NIHSS** | 17 (12, 22) | 17 (13, 22) | >0.9 |
| **CSC NIHSS** | 18 (13, 23) | 17 (11, 21) | **0.006** |
| **Delta NIHSS** | 0 (-2, 3) | -1 (-6, 2) | **0.004** |
| **OSH ASPECTS** | 8.84(1.29) | 9.19(1.05) | **0.016** |
| **24h NIHSS** | 12 (6, 19) | 9 (4, 17) | **0.022** |
| **Time to angio suite** | 4h45min (3h10min, 7h53min) | 3h30min (2h40min, 4h28) | **<0.001** |
| **LVO on CTA** | 191 (86%) | 127 (69%) | **<0.001** |
| **M1 occlusion** | 86 (45%) | 71 (55%) | 0.087 |
| **M2M3 occlusion** | 33 (17%) | 17 (13%) | 0.3 |
| **ICA terminus occlusion** | 36 (19%) | 26 (20%) | 0.8 |
| **Basilar occlusion** | 25 (13%) | 10 (7.8%) | 0.13 |
| **LVO confirmed by DSA** |  |  | **<0.001** |
| *No* | 11 (4.9%) | 36 (20%) |  |
| *Yes* | 212 (95%) | 144 (80%) |  |
| *Yes (done two days later)* | 0 (0%) | 1 (0.6%) |  |
| **Groin puncture** | 14 (100%) | 35 (80%) | 0.10 |
| **Thrombectomy performed** | 213 (96%) | 142 (77%) | **<0.001** |
| **Reason for no EVT** |  |  | 0.4 |
| *distal occlusion* | 3 (30%) | 18 (43%) |  |
| *exam improved* | 1 (10%) | 11 (26%) |  |
| *patent vessels* | 5 (50%) | 11 (26%) |  |
| *subocclusive* | 1 (10%) | 2 (4.8%) |  |
| **Discharge Facility** |  |  | 0.072 |
| *Death* | 43 (19%) | 27 (15%) |  |
| *Home* | 34 (15%) | 45 (24%) |  |
| *Hospice* | 17 (7.7%) | 8 (4.3%) |  |
| *IPR* | 105 (47%) | 92 (50%) |  |
| *LTF* | 1 (0.5%) | 1 (0.5%) |  |
| *SNF* | 22 (9.9%) | 11 (6.0%) |  |
| **30-day mRS** |  |  | 0.14 |
| *0* | 7 (6.9%) | 12 (12%) |  |
| *1* | 10 (9.8%) | 20 (20%) |  |
| *2* | 11 (11%) | 16 (16%) |  |
| *3* | 17 (17%) | 11 (11%) |  |
| *4* | 15 (15%) | 12 (12%) |  |
| *5* | 4 (3.9%) | 3 (3.0%) |  |
| *6* | 38 (37%) | 26 (26%) |  |
| **90-day mRS** |  |  | 0.4 |
| *0* | 8 (5.9%) | 11 (9.3%) |  |
| *1* | 15 (11%) | 22 (19%) |  |
| *2* | 15 (11%) | 17 (14%) |  |
| *3* | 18 (13%) | 14 (12%) |  |
| *4* | 16 (12%) | 14 (12%) |  |
| *5* | 4 (3.0%) | 3 (2.5%) |  |
| *6* | 59 (44%) | 37 (31%) |  |
| ^1^Median (Q1, Q3); Mean(SD); n (%) | | | |
| ^2^Wilcoxon rank sum test; Pearson's Chi-squared test; Fisher's exact test | | | |

Table S3. Clinical Characteristics and Functional Outcomes of Patients Receiving Versus Not Receiving Intravenous Thrombolytics
